# Expression of Axonal Transport Proteins in Dopaminergic Neurons of the Substantia Nigra in Mouse Models of Preclinical and Clinical Stages of Parkinson’s Disease

**DOI:** 10.3390/ijms27114895

**Published:** 2026-05-28

**Authors:** Anna Kolacheva, Dmitry Troshev, Alyona Antonova, Ekaterina Pavlova, Vsevolod Bogdanov, Varvara Kalashnikova, Anna Popova, Maria Shchepina, Michael Ugrumov

**Affiliations:** 1 Laboratory of Neural and Neuroendocrine Regulations, Koltzov Institute of Developmental Biology, Russian Academy of Sciences, 26 Vavilova Street, 119334 Moscow, Russiamichael.ugrumov@mail.ru (M.U.); 2 The Institute of Digital Biodesign and Artificial Intelligence in Medicine, I.M. Sechenov First Moscow State Medical University of the Ministry of Health of the Russian Federation, 8-2 Trubetskaya Street, 119991 Moscow, Russia

**Keywords:** Parkinson’s disease, preclinical and clinical stages, MPTP, dopaminergic neurons, axonal transport, kinesin, dynein, microtubule

## Abstract

Impairment of axonal transport may contribute to the degeneration of dopaminergic (DAergic) neurons in the substantia nigra (SN), a key event in Parkinson’s disease (PD) pathogenesis. Due to the lack of early diagnosis, changes in axonal transport at the preclinical stage can only be studied in PD models. We assessed gene expression (RT-PCR after cell sorting) and protein levels (semiquantitative immunohistochemistry) of axonal transport-related proteins in SN DAergic neurons from mice in subchronic MPTP models of PD (preclinical and clinical stages) and controls. The proteins studied included α-tubulin (*Tuba1a*), β-tubulin (*Tubb3*), kinesin (*Kif5b*, *Klc1*), dynein (*Dynll1*, *Dync1i1*), dynactin (*Dctn1*), microtubule affinity-regulating kinase 1 (*Mark1*), and tau (*Mapt*). In the preclinical stage, *Kif5b* expression and Kif5B level were increased, possibly to compensatorily preserve anterograde transport. *Dynll1* and *Tuba1a* were upregulated, whereas *Dync1i1* and *Mapt* were downregulated, with no change in tubulin or tau protein levels. In the clinical stage, *Klc1*, *Dync1i1*, *Dctn1*, *Mark1*, and *Mapt* expression and Kif5B protein levels decreased. These data indicate that transcriptional alterations in axonal transport proteins precede protein-level changes in DAergic neurons. The upregulation of Kif5B in the preclinical stage suggests that axonal transport proteins may serve as potential early therapeutic targets in PD.

## 1. Introduction

Parkinson’s disease (PD) is a neurodegenerative disorder characterized by severe motor impairments resulting in patient disability [1,2]. For decades, the disease develops at the preclinical stage without specific motor symptoms [3]. This makes it impossible to diagnose PD over a long period of its development and, therefore, to study the mechanisms of pathogenesis—neurodegeneration and neuroplasticity—in patients at the preclinical stage.

A milestone in the pathogenesis of PD is the degradation of the nigrostriatal dopaminergic (DAergic) system of the brain [4]. By the time motor symptoms first appear and the disease is diagnosed, approximately 50% of DAergic neurons are lost in the SN, and the levels of dopamine (DA), tyrosine hydroxylase, and DA transporter are reduced by 70% in the caudate nucleus and/or putamen compared to age-matched controls [5,6]. In PD, degradation of nigrostriatal DAergic neurons is believed to begin at the axon terminals and then spread retrogradely to the neuronal cell bodies [7,8].

It is suggested that one of the main causes of degradation of DAergic axon terminals is a disruption of axonal transport, which normally ensures the delivery of organelles and proteins from neuronal cell bodies [9]. Axonal transport is provided by the coordinated functioning of three classes of proteins: (i) structural proteins that form microtubules (α- and β-tubulins); (ii) motor proteins, kinesins and dynein, which mediate anterograde and retrograde transport, respectively; and (iii) regulatory proteins, such as tau protein, that stabilize microtubules, and adaptor proteins that link vesicles and organelles to motor proteins.

Postmortem studies of patients with PD have shown that gene expression and levels of axonal transport proteins in DAergic neurons differ compared to age-matched controls [10,11,12,13,14,15,16,17,18,19,20]. As the disease progresses, the list of altered axonal transport proteins expands. Specifically, at the early clinical stage of PD, DAergic neurons are characterized by reduced levels of anterograde motor proteins (kinesins), whereas disease progression is associated with a subsequent decline in retrograde motor proteins (dynein) [11]. However, previous studies conducted in animal models of PD and in postmortem tissue of patients failed to ascertain at what stage of PD the metabolism of axonal transport proteins changes. In other words, it is necessary to clarify whether the metabolism of axonal transport proteins changes only at the clinical stage, as already described in the literature [10,11,12,13,14,15,16,17,18,19,20], or whether this occurs earlier—at the preclinical (asymptomatic) stage.

In the absence of a preclinical diagnosis of PD, this question can only be addressed using experimental models of the progressive development of this disease. Therefore, the aim of this study was to evaluate gene expression and synthesis of axonal transport proteins in DAergic neurons of the SN in subchronic mouse models of preclinical and clinical PD induced by 1-methyl-4-phenyl-1,2,3,6-tetrahydropyridine (MPTP), which we developed previously [21]. The solution to this problem will open up broad prospects for the development of pathogenetic neuroprotective therapy aimed at prolonging the preclinical (asymptomatic) stage of PD.

## 2. Results

### 2.1. Validation of Mouse Subchronic Models of Parkinson’s Disease at Preclinical and Clinical Stages Used in This Study

**Figure 1:**
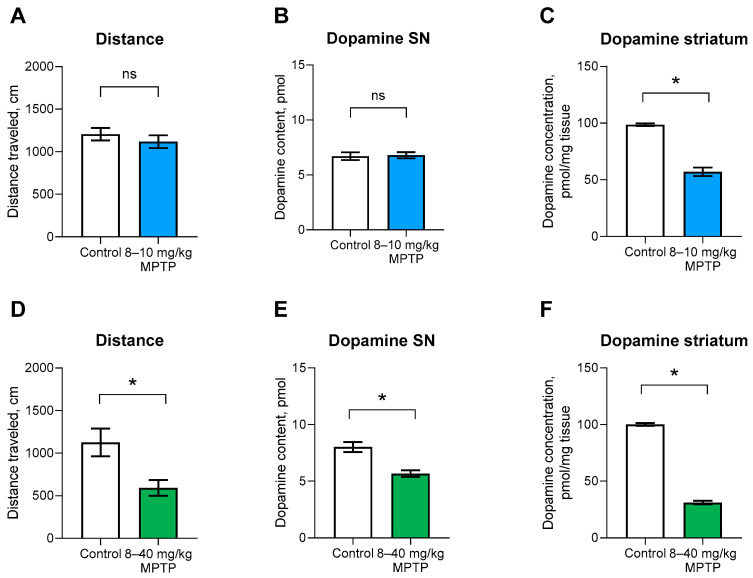
Open field distance traveled for 6 min by mice (**A**,**D**), as well as substantia nigra (SN) dopamine content (**B**,**E**) and striatum dopamine concentration (**C**,**F**) (*n* = 7–11) 24 h after subcutaneous administration of 1-methyl-4-phenyl-1,2,3,6-tetrahydropyridine (MPTP) at doses of 8 and 10 mg/kg with a 24 h interval between the injections (**A**–**C**), and after subcutaneous administration of MPTP at doses of 8, 10, 12, 16, 20, 26, and 40 mg/kg at 24 h intervals between the injections (**D**–**F**). The control groups were injected with saline according to the same regimens. The groups were tested for normality in distribution using the Shapiro–Wilk test. Statistical analysis was performed using the unpaired *t*-test or Mann–Whitney test (* *p* ≤ 0.05; ns, not significant) in comparison of experimental and control groups. The data are presented as mean ± SEM.

In the mouse model of the preclinical stage of PD, no changes in the distance traveled in the open field test were detected 24 h following administration of 8–10 mg/kg MPTP (Figure 1A). Also, DA content in the SN remained unchanged (Figure 1B), while DA concentration in the striatum decreased by 42% (*p* < 0.0001) (Figure 1C).

In the model of the clinical stage of PD, the traveled distance of mice 24 h after administration of 8–40 mg/kg MPTP was reduced by 47% (*p* = 0.0084) (Figure 1D). Also, DA content in the SN (Figure 1E) and its concentration in the striatum (Figure 1F) decreased by 29% (*p* = 0.0002) and 69% (*p* < 0.0001), respectively.

### 2.2. Gene Expression of Axonal Transport Proteins in Substantia Nigra Tissue in Mouse Models of Parkinson’s Disease at Preclinical and Clinical Stages

**Figure 2:**
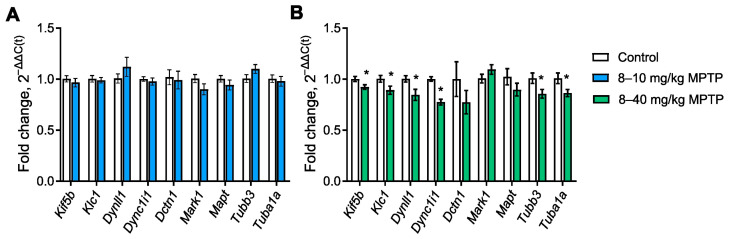
Gene expression in the substantia nigra tissue of mice 24 h after subcutaneous administration of 1-methyl-4-phenyl-1,2,3,6-tetrahydropyridine (MPTP) under two regimens: (**A**) doses of 8 and 10 mg/kg with a 24 h interval between injections; (**B**) doses of 8, 10, 12, 16, 20, 26, and 40 mg/kg with 24 h intervals between injections. Control groups received saline following the same regimens. Normality of distribution was tested using the Shapiro–Wilk test. Statistical analysis was performed using the unpaired *t*-test (* *p* ≤ 0.05) for comparison between experimental and control groups. Data are presented as mean ± SEM. *n* = 8. *Kif5b*, kinesin family member 5B gene; *Klc1*, kinesin light chain 1 gene; *Dynll1*, dynein light chain 1 gene; *Dync1i1*, cytoplasmic dynein 1 intermediate chain 1 gene; *Dctn1*, dynactin 1 gene; *Mark1*, microtubule affinity regulating kinase 1 gene; *Mapt*, microtubule-associated protein tau gene; *Tubb3*, β3-tubulin gene; *Tuba1a*, α-tubulin gene.

The expression of all axonal transport protein genes studied—*Kif5b* (kinesin family member 5B gene), *Klc1* (kinesin light chain 1 gene), *Dynll1* (dynein light chain 1 gene), *Dync1i1* (cytoplasmic dynein 1 intermediate chain 1 gene), *Dctn1* (dynactin 1), *Mark1* (microtubule affinity regulating kinase 1 gene), *Mapt* (microtubule-associated protein tau gene), *Tubb3* (tubulin beta 3 class III, further β3-tubulin, gene), and *Tuba1a* (tubulin alpha 1a, further α-tubulin, gene)—was shown to remain unchanged in the SN tissue in a model of preclinical PD (Figure 2A).

In a model of clinical PD, the expression of 6 of the 8 genes studied in SN tissue was reduced as follows: *Kif5b* by 7.9% (*p* = 0.0344), *Klc1* by 11.1% (*p* = 0.0497), *Dynll1* by 15.6% (*p* = 0.0258), *Dync1i1* by 22.6% (*p* < 0.0001), *Tubb3* by 15.1% (*p* = 0.0389), and *Tuba1a* by 14.3% (*p* = 0.038) (Figure 2B).

### 2.3. Gene Expression of Axonal Transport Proteins in Sorted Dopaminergic Neurons in Mouse Models of Parkinson’s Disease at Preclinical and Clinical Stages

**Figure 3:**
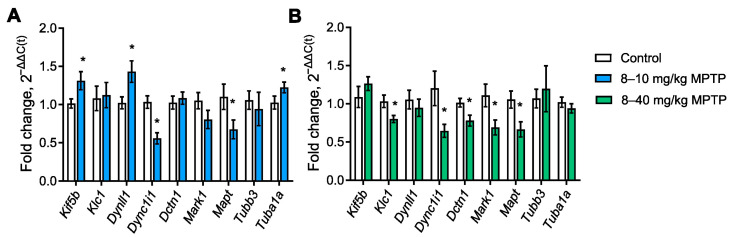
Gene expression in sorted dopaminergic neurons of the substantia nigra in mice 24 h after subcutaneous administration of 1-methyl-4-phenyl-1,2,3,6-tetrahydropyridine (MPTP) under two regimens: (**A**) doses of 8 and 10 mg/kg with a 24 h interval between injections; (**B**) doses of 8, 10, 12, 16, 20, 26, and 40 mg/kg with 24 h intervals between injections. Control groups received saline following the same regimens. Normality of distribution was tested using the Shapiro–Wilk test. Statistical analysis was performed using the unpaired *t*-test (* *p* ≤ 0.05) for comparison between experimental and control groups. Data are presented as mean ± SEM; *n* = 9–10. *Kif5b*, kinesin family member 5B gene; *Klc1*, kinesin light chain 1 gene; *Dynll1*, dynein light chain 1 gene; *Dync1i1*, cytoplasmic dynein 1 intermediate chain 1 gene; *Dctn1*, dynactin 1 gene; *Mark1*, microtubule affinity regulating kinase 1 gene; *Mapt*, microtubule-associated protein tau gene; *Tubb3*, β3-tubulin gene; *Tuba1a*, α-tubulin gene.

In the preclinical stage of PD, in DAergic neurons isolated from the SN of mice, the expression of *Kif5b*, *Dynll1* and *Tuba1a* increased by 29.3% (*p* = 0.0457), 40% (*p* = 0.025) and 23.2% (*p* = 0.0446), respectively, compared with the expression of these genes in DAergic neurons from the control animals. Meanwhile, the expression of *Dync1i1* decreased by 46% (*p* = 0.0005) and *Mapt* decreased by 45.5% (*p* = 0.0234) (Figure 3A). The expression of *Dctn1*, *Tubb3* and *Mark1* was unchanged.

The gene expression in the sorted SN DAergic neurons of mice when modeling the clinical stage of PD differed significantly from the gene expression in the SN tissue. Thus, a significant decrease in the expression of several genes was observed in SN DAergic neurons: *Klc1* by 22.3% (*p* = 0.024), *Dync1i1* by 46.3% (*p* = 0.0331), *Dctn1* by 23.1% (*p* = 0.0194), *Mark1* by 37.8% (*p* = 0.0302), and *Mapt* by 37.1% (*p* = 0.0172), whereas the expression levels of *Kif5b*, *Tubb3*, *Tuba1a*, and *Dynll1* did not differ significantly (Figure 3B).

### 2.4. The Content of Axonal Transport Proteins in Cell Bodies of Dopaminergic Neurons in the Substantia Nigra in a Mouse Model of Parkinson’s Disease at Preclinical Stage

**Figure 4:**
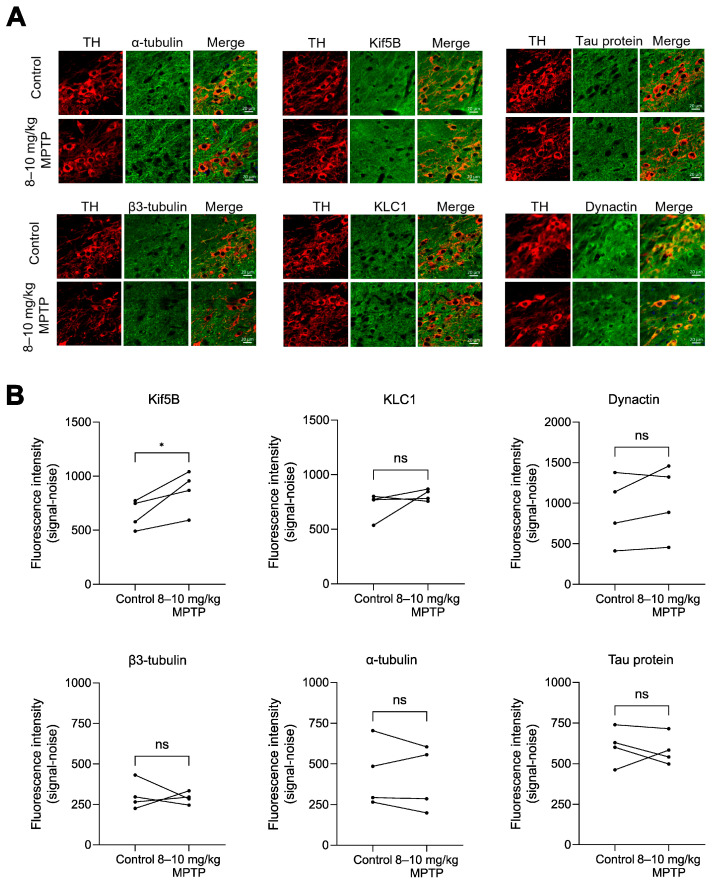
(**A**) Tyrosine hydroxylase-immunoreactive neurons (cell bodies, red) in the substantia nigra immunostained for kinesin family member 5B (Kif5B), kinesin light chain 1 (KLC1), dynactin, β3-tubulin, α-tubulin or tau protein (green) in mice 24 h after subcutaneous administration of 1-methyl-4-phenyl-1,2,3,6-tetrahydropyridine (MPTP) at doses of 8 and 10 mg/kg, with a 24 h interval between the injections (8–10 mg/kg MPTP). The control groups were injected with saline using the same regimens (Control). Scale bar: 20 µm. (**B**) The content of Kif5B, KLC1, dynactin, β3-tubulin, α-tubulin, and tau protein in cell bodies of tyrosine hydroxylase-immunoreactive neurons of the substantia nigra (*n* = 4) in mice 24 h after subcutaneous administration of MPTP at doses of 8 and 10 mg/kg, with a 24 h interval between the injections (8–10 mg/kg MPTP) or saline (Control). The groups were compared for conformity to the normal distribution using the Shapiro-Wilk test. Statistical analysis was performed using the paired *t*-test or Wilcoxon matched-pairs signed rank test (* *p* ≤ 0.05; ns, not significant) for comparison of the experimental and control groups.

Kif5B protein levels increased by 34% (*p* = 0.0451) in DAergic neurons (cell bodies) of the SN in a mouse model of preclinical PD, whereas KLC1, dynactin, β3-tubulin, α-tubulin and tau protein levels were unchanged in these neurons (Figure 4A,B).

### 2.5. The Content of Axonal Transport Proteins in Cell Bodies of Dopaminergic Neurons in the Substantia Nigra in a Mouse Model of Parkinson’s Disease at Clinical Stage

**Figure 5:**
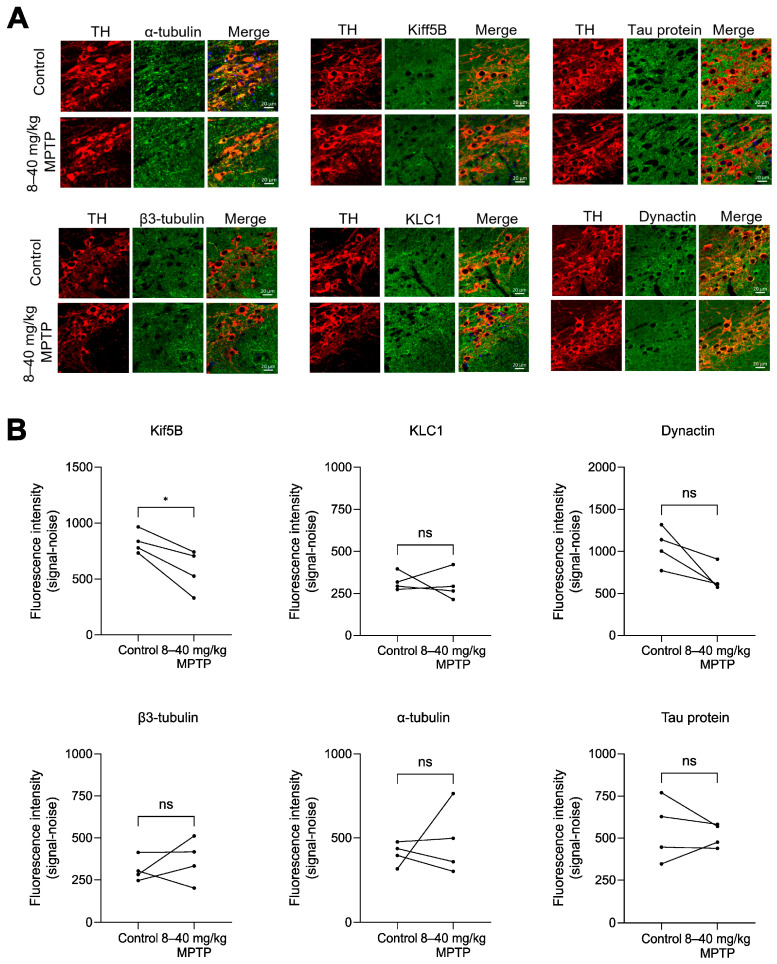
(**A**) Tyrosine hydroxylase-immunoreactive neurons (cell bodies, red) in the substantia nigra immunostained for kinesin family member 5B (Kif5B), kinesin light chain 1 (KLC1), dynactin, β3-tubulin, α-tubulin or tau protein (green) in mice 24 h after subcutaneous administration of 1-methyl-4-phenyl-1,2,3,6-tetrahydropyridine (MPTP) at doses of 8, 10, 12, 16, 20, 26 and 40 mg/kg, with a 24 h interval between the injections (8–40 mg/kg MPTP). The control groups were injected with saline using the same regimens (Control). Scale bar: 20 µm. (**B**) The content of Kif5B, KLC1, dynactin, β3-tubulin, α-tubulin, and tau protein in the cell bodies of tyrosine hydroxylase-immunoreactive neurons of the substantia nigra (*n* = 4) in mice 24 h after subcutaneous administration of MPTP at doses of 8, 10, 12, 16, 20, 26 and 40 mg/kg, with a 24 h interval between the injections (8–40 mg/kg MPTP) or saline (Control). The groups were compared for conformity to the normal distribution using the Shapiro-Wilk test. Statistical analysis was performed using the paired *t*-test or Wilcoxon matched-pairs signed rank test (* *p* ≤ 0.05; ns, not significant) for comparison of the experimental and control groups.

In a mouse model of clinical PD, the content of Kif5B decreased by 32% (*p* = 0.0207) in DAergic neurons of the SN (Figure 5A,B). The content of KLC1, tau protein, β3-tubulin, α-tubulin and dynactin remained unchanged in DAergic neurons of the SN (Figure 5A,B).

## 3. Discussion

It is believed that axonal transport impairment may contribute to the degeneration of nigrostriatal DAergic neurons in PD, and these alterations may begin as early as the preclinical stage of the disease [9]. Most studies investigating axonal transport in PD have been performed on postmortem tissue obtained from patients many years after diagnosis [13,15,16,17,18]. Although treatment details are not provided in these studies, it is reasonable to assume that the patients had received long-term 3-(3,4-dihydroxyphenyl)-L-alanine (L-DOPA) and/or DA receptor agonist therapy [22]. This is an important consideration, as such treatment may itself affect axonal transport. For example, activation of D2 receptors inhibits serine/threonine protein kinase B (Akt), which normally suppresses the activity of glycogen synthase kinase-3β (GSK-3β). As a result of reduced Akt activity, GSK-3β remains active [23,24], which is shown in postmortem SN tissue of PD patients [25]. One of the GSK-3β targets is tau protein; phosphorylation of tau leads to microtubule destabilization and disrupts the binding of kinesin to microtubules, thereby potentially altering axonal transport [26,27,28]. As a result, long-term antiparkinsonian therapy itself may modify axonal transport in DAergic neurons of patients.

It should be noted that in many studies based on postmortem material, the expression of genes and the levels of axonal transport proteins were assessed in SN tissue, which has a heterogeneous cellular composition [10,16,29,30]. This approach precludes the targeted assessment of these parameters specifically within DAergic neurons. Moreover, in the context of neurodegeneration, it predominantly reflects changes in the overall cellular composition of the tissue [31]. Therefore, the usage of clinical material has substantial limitations that complicate the investigation of pathogenesis and compensatory mechanisms at the clinical stage of PD. Concurrently, the lack of early diagnostic tools prevents the study of these processes during the preclinical stage of the disease.

Taking these factors into account, we used a subchronic PD model in mice to investigate the molecular mechanisms underlying axonal transport impairment in DAergic neurons in PD. This model reproduces the progressive degeneration of the nigrostriatal system [21]. Gene expression was analyzed in sorted DAergic neurons of the nigrostriatal system of mice using a previously developed method [32]. Protein levels in the cell bodies of these neurons were assessed by semiquantitative immunohistochemistry. When comparing our data obtained in the clinical stage PD model with data derived from postmortem material from PD patients [12,13,17,18,19,20], we were guided by three underlying assumptions. First, we assumed that the alterations in gene expression and levels of axonal transport proteins observed in PD patients are associated with neurodegenerative processes in the nigrostriatal system, rather than with symptomatic treatment. Second, we predominantly relied on data from studies performed on isolated DAergic neurons of the SN [17,18,19,20], and only in the absence of such target-specific data did we refer to studies conducted on whole SN tissue homogenates [13]. Third, we interpret our findings as indicating concurrent changes in the functional parameters of the nigrostriatal system (DA content), together with changes in the expression of genes encoding axonal transport proteins and in the levels of these proteins in DAergic neurons in a model of PD.

### 3.1. Validation of the Subchronic Parkinson’s Disease Model

First, we validated the subchronic mouse models of the preclinical and clinical stages of PD (MPTP administration in doses of 8–10 mg/kg and 8–40 mg/kg, respectively) by evaluating the distance traveled by mice in the open field test and analyzing DA levels in the striatum and SN. After sequential MPTP injections at escalating doses of 8 and 10 mg/kg, DA levels decreased exclusively in the striatum, whereas DA levels in the SN and the animals’ motor function remained unaffected. In contrast, MPTP administration at increasing doses ranging from 8 to 40 mg/kg induced motor impairments accompanied by DA depletion in both the striatum and the SN. These findings confirmed that MPTP administration according to the 8–10 mg/kg and 8–40 mg/kg regimens reproduces the preclinical and clinical stages of PD, respectively [21].

### 3.2. Alterations in the Gene Expression and Protein Content of α- and β-Tubulin

**Figure 6:**
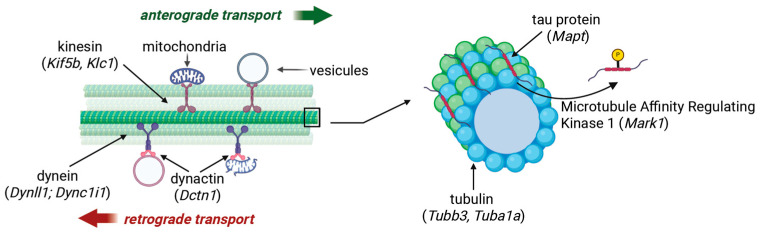
Schematic representation of microtubule organization (α/β-tubulin heterodimers), motor proteins (kinesin, dynein, and the associated dynactin), and regulatory proteins (tau protein, microtubule affinity-regulating kinase 1) that mediate axonal transport. The names of the genes encoding the respective proteins are shown in brackets. Created in BioRender. Blokhin, V. (2026) https://BioRender.com/6eewzi1.

A crucial element of the cytoskeleton responsible for the transport of vesicles and cellular organelles is the microtubule, which consists of an α- and β-tubulin heterodimer (Figure 6). Microtubules undergo constant remodeling [33], and their stability is regulated by post-translational modifications, such as the acetylation of α-tubulin, or by interaction with the tau protein [34]. Recent studies have shown that the redistribution of acetylated α-tubulin is associated with α-synuclein accumulation in the brains of patients with PD and appears to be linked to Lewy body formation [35]. Tau protein itself can be phosphorylated, which promotes its dissociation from microtubules, thereby reducing microtubule stability [36,37].

Our findings demonstrate that in a model of the clinical stage of PD, the expression of *Tubb3* and *Tuba1a* was downregulated in the SN tissue; however, their expression remained unchanged within the cell bodies of DAergic neurons of the SN. A study by Tiklová performed on neuromelanin-containing neurons (hereafter referred to as DAergic neurons) isolated from the postmortem SN of patients at early stages of PD (Hoehn and Yahr stages 1–2) reported no alterations in the expression of these genes [19]. Conversely, several studies investigating the later stages of PD have observed a downregulation in *Tubb3* and *Tuba1a* expression within DAergic neurons [17,18,20]. Taken together, these findings suggest that the dysregulation of genes encoding microtubule proteins becomes more pronounced with disease progression.

According to our data, the levels of α-tubulin and β3-tubulin proteins in sorted DAergic neurons of the SN were unchanged in the clinical stage model of PD compared to the control. The absence of alterations in α-tubulin levels has been previously demonstrated both in postmortem SN tissue of PD patients [12] and in experimental models of the disease [38]. Furthermore, the content of acetylated α-tubulin, which plays a role in microtubule stabilization, remained unchanged in an in vivo mouse model of PD [38] but was reduced in the SN tissue of PD patients [12]. However, when interpreting data derived from SN tissue, it is crucial to consider, as previously mentioned, that the SN comprises a heterogeneous cell population [39]. Therefore, the previously reported reduction in acetylated α-tubulin levels may not reflect altered metabolism within DAergic neurons, but rather generalized tissue degeneration during disease progression that affects multiple brain structures [40].

In the preclinical stage model of PD, we observed an upregulation of *Tuba1a* expression, which, however, was not accompanied by changes in α-tubulin protein levels within the cell bodies of SN DAergic neurons. Similarly, *Tubb3* expression and β3-tubulin content in DAergic neurons remained at control levels in this model. The absence of changes in tubulin content in the cell bodies of neurons does not guarantee preservation of these proteins’ levels or of the microtubules’ stability in the axons. It is well known that the axonal compartment of DAergic neurons is highly vulnerable. Indeed, in cultured midbrain DAergic neurons, complete axonal microtubule breakdown, accompanied by a loss of the acetylated α-tubulin signal, has been shown to occur as early as 24 h after toxin exposure [41,42,43]. Thus, even while microtubule protein levels are preserved in the neuronal cell bodies, pathological changes may develop independently within the axons at earlier stages of the disease. This aligns with clinical data demonstrating a more pronounced loss of axons within the striatum compared to the loss of DAergic neuron cell bodies in the SN of PD patients [3,44]. In the future, it is crucial to characterize the state of microtubules in the axons of DAergic neurons—including tubulin levels and their post-translational modifications—while studying DAergic neuron degeneration in vivo.

### 3.3. Alterations in Gene Expression and tau Protein Levels

As mentioned earlier, tau protein plays a pivotal role in microtubule stabilization (Figure 6). Its phosphorylation induces its dissociation from microtubules, thereby reducing their stability [26,45]. Furthermore, the phosphorylated form of tau exhibits a higher affinity for kinesin [26]. Finally, the accumulation of phosphorylated tau promotes its aggregation, and its co-localization with α-synuclein in Lewy bodies in PD indicates a potential contribution of tau pathology to disease progression [46].

According to our data in the clinical stage model of PD, tau gene (*Mapt*) expression didn’t change in the SN tissue but was reduced in DAergic neurons of the SN. Notably, no alterations in *MAPT* expression levels were found in the cell bodies of DAergic neurons of the SN in postmortem material from PD patients at Hoehn–Yahr stages 2–3 [19]. However, during disease progression, MAPT expression declines not only in DAergic neurons [19,20] but also in the whole SN tissue [13,16].

Such a downregulation of *MAPT*, observed in both experimental models and postmortem tissues, can be postulated as a compensatory mechanism. Given that microtubule-unbound phosphorylated tau is capable of forming toxic oligomers [47], the reduction in *MAPT* transcription enables the cell to deplete the pool of substrate available for pathological posttranslational modifications and subsequent aggregation.

However, data on tau levels in SN DAergic neurons and in SN tissue from postmortem PD material and from PD models remain contradictory, with reports of either no change or an increase [12,14,20,48,49]. Despite these discrepancies, a much more consistent pattern emerges in relation to phosphorylated tau, which is elevated compared to control subjects [12,48,49]. This may indicate that the key factor in PD pathogenesis is not necessarily an alteration in tau content, but rather a shift toward pathologically modified forms of the protein.

Downregulation of *Mapt* expression in DAergic neurons of the SN occurs as early as the preclinical model of PD. However, consistent with the model of the clinical stage, this does not lead to changes in tau content within the cell bodies of DAergic neurons of the SN. Given that similar changes have been demonstrated in the clinical stage model of PD, and that dysregulation of tau post-translational modifications may already occur at early stages of DAergic neuron degeneration, the reduction in *Mapt* expression in a model of the preclinical stage may be interpreted as a compensatory mechanism. As noted above, tau is phosphorylated by GSK-3β, whose activity is upregulated in PD [25]. Inhibition of GSK-3β preserves microtubule integrity in DAergic neurons upon MPTP treatment, indicating that GSK-3β is a potential therapeutic target for neuroprotection in PD [27,50].

### 3.4. Alterations in the Expression and Levels of Motor Proteins Involved in Anterograde Axonal Transport

Kinesin-1 is a heterotetramer composed of heavy chains (KHC or Kif5) and light chains (KLC) (Figure 6). Kif5B, one of the Kif protein family members, translocates along microtubules through ATP hydrolysis [51]. The C-terminal domain of Kif5B folds back to inhibit the motor domain, thereby maintaining kinesin-1 in an inactive, autoinhibited state. This autoinhibition is relieved when the KLC1 binds to target vesicles or organelles, subsequently leading to kinesin activation [52].

In the clinical stage model of PD, we found that *Kif5B* expression in DAergic neurons of the SN remained unchanged, whereas Kif5B protein levels in the cell bodies of DAergic neurons were reduced. Similar alterations have been previously reported in DAergic neurons of the postmortem SN tissue of PD patients [11,17,18]. If Kif5B levels are also decreased within the axons of DAergic neurons, this could indicate a decline in the amount of transported cargo. Furthermore, microtubule instability—for example, following the tau protein dissociation—likely contributes to a reduction in average transport velocity. This aligns with findings from studies on DAergic neuron cultures and *Danio rerio* models, which demonstrated not only a slowing of anterograde transport but also an increase in the number of “stationary” cargoes [42,43,53,54].

Notably, in the preclinical stage model of PD, increased *Kif5B* expression and Kif5B protein levels were observed in DAergic neurons of the SN. At the same time, the gene expression and protein content of KLC1 in these neurons remained unchanged. Given that KLC1 is required for the transport of Kif5B [55,56], assessing their stoichiometric ratio in DAergic neurons is crucial. To this end, we used publicly available proteomic data from the cell bodies in the SN, axons in the medial forebrain bundle, and arborized axons with varicosities in the striatum of mouse SN DAergic neurons [57]. Normalizing to alpha-tubulin (Tuba4a, P68368) as an internal standard revealed that the KLC1:Kif5B ratio in DAergic neurons at the level of cell bodies, axons, and varicosities was 2:1, 3:1, and 6:1, respectively. Thus, KLC1 prevails over Kif5B in DAergic neurons; therefore, Kif5B level is a limiting factor for axonal transport. Consequently, the upregulation of *Kif5B* gene expression and protein levels at the model of the preclinical stage of PD can be interpreted as a compensatory mechanism aimed at maintaining anterograde axonal transport.

Moreover, upregulating *Kif5B* expression may be considered a potentially effective strategy for restoring the axonal transport of essential cargoes, including mitochondria, in PD. However, implementing this approach necessitates altering a strict balance between enhancing the transport efficiency and preserving the structural integrity of microtubules. Failure to maintain this equilibrium could result in the pathological accumulation of organelles within axonal varicosities, thereby driving the progression of neurodegeneration.

### 3.5. Alterations in the Gene Expression and Content of Motor Proteins Involved in Retrograde Axonal Transport

In contrast to the multiple isoforms of kinesin, dynein is the sole protein complex responsible for the retrograde transport of endosomes, autophagosomes, and mitochondria within neurons [58,59] (Figure 6). Its heavy chain comprises a tail region required for dimerization and a motor domain that moves along microtubules via ATP hydrolysis, whereas the light and intermediate chains bind vesicles and organelles. For directed movement along microtubules, dynein must assemble into a functional complex with dynactin and adaptor proteins [60].

It has been established that in the clinical stage model of PD, decreased mRNA levels of *Dync1i1* and *Dctn1* are observed in SN DAergic neurons, whereas *Dynll1* expression remains stable. At the same time, dynactin levels in DAergic neurons remain unchanged. Overall, these data are generally consistent with findings from postmortem SN tissue from PD patients [10,15,17,18,20]. Previous research has shown that the expression of genes encoding dynein light and intermediate chains (*DYNLL1*, *DYNC1I1*) in the SN DAergic neurons is decreased even at the early clinical stage of PD [19]. However, the protein levels of the corresponding dynein chains in SN DAergic neurons of patients at early clinical stages of PD (Hoehn and Yahr stages 1–2) show no significant alterations [11,14,20]. Furthermore, despite conflicting data on *DCTN1* expression in DAergic neurons of PD patients, dynactin levels also appear to remain unchanged [12,18,20].

The obtained data and literature indicate that disturbances of the retrograde transport system initially develop at the transcriptomic level and not at the level of protein changes. This conclusion is supported by data obtained from the model of the preclinical stage of PD. At this stage, we observed multidirectional changes in *Dynll1* and *Dync1i1* expression, with no alterations in *Dctn1* transcript levels. Concurrently, consistent with the model of the clinical stage, dynactin protein levels remained unchanged.

Therefore, as the nigrostriatal system degenerates in PD, changes in motor proteins in SN DAergic neurons first manifest at the mRNA level, whereas the decline in protein content occurs later, at more advanced stages of the disease.

## 4. Materials and Methods

### 4.1. Animals

Male C57BL/6 mice (*n* = 93) aged 8–12 weeks and weighing 20–25 g, obtained from the Stolbovaya breeding center (SCBMT RAMS, Stolbovaya, Moscow reg., Russia) were used in this study. The animals were kept at 22 ± 1 °C, with a 12 h day/night cycle and free access to food and water. Experimental procedures were performed according to the *National Institute of Health Guide for the Care and Use of Laboratory Animals* (8th edition, 2011) regulations and were approved by the Animal Care and Use Committee of the Koltzov Institute of Developmental Biology of the Russian Academy of Sciences (protocol No. 95 from 26 June 2025).

### 4.2. Experiments

#### 4.2.1. Reproduction of a Subchronic Model of Parkinson’s Disease

Two days before modeling the preclinical and clinical stages of PD using MPTP (Sigma-Aldrich, St. Louis, MO, USA), the distance traveled by the mice over a 6 min period was recorded using an automated PhenoMaster device (TSE Systems, Berlin, Germany) with TSE PhenoMaster V5.7.9 software. The mice were then divided into groups based on the distance traveled, ensuring that the mean distance traveled was the same across groups.

**Figure 7:**
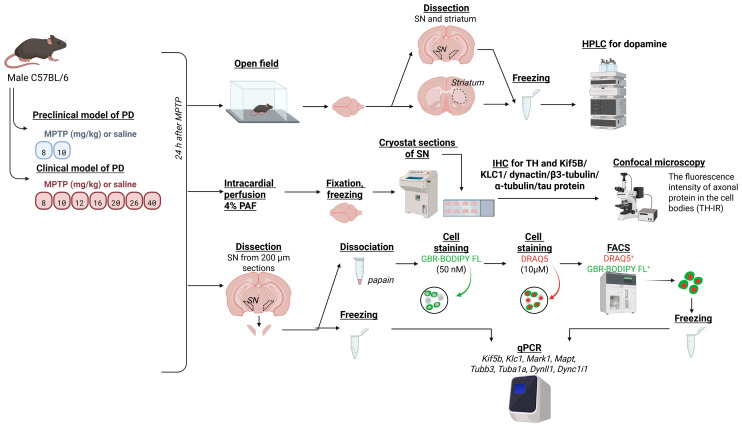
Schematic representation of experiments to reproduce a subchronic mouse model of Parkinson’s disease induced by subcutaneous injection of 1-methyl-4-phenyl-1,2,3,6-tetrahydropyridine (MPTP) to mice at doses of 8 and 10 mg/kg (preclinical stage) or 8, 10, 12, 16, 20, 26, and 40 mg/kg (clinical stage), with a 24 h interval between the injections, and to assess gene expression and the content of respective axonal transport proteins in dopaminergic neurons of the substantia nigra in this model of Parkinson’s disease. Created in BioRender. Blokhin, V. (2026) https://BioRender.com/6eewzi1. DRAQ5, 1,5-bis{[2-(di-methylamino)ethyl]amino}-4,8-dihydroxyanthracene-9,10-dione, a cell-permeable DNA dye (far-red fluorescence); GBR-BODIPY FL, an analogue of the dopamine reuptake inhibitor GBR12909 containing the fluorophore BODIPY^®^ FL (green fluorescence); HPLC, high-performance liquid chromatography; IHC, immunohistochemistry; Kif5B, kinesin family member 5B; KLC1, kinesin light chain 1; MPTP, 1-methyl-4-phenyl-1,2,3,6-tetrahydropyridine; PAF, paraformaldehyde; PD, Parkinson’s disease; SN, substantia nigra; TH, tyrosine hydroxylase.

To model the preclinical and clinical stages of PD, the animals were subcutaneously injected with MPTP according to a previously developed protocol, which induces the progressive degradation of the nigrostriatal DAergic system [21] (Figure 7). To model the preclinical stage of PD, the animals were injected with MPTP at doses of 8 and 10 mg/kg, with a 24 h interval between the injections (*n* = 10) (hereafter denoted as 8–10 mg/kg MPTP). To model the clinical stage of PD, MPTP was administered at increasing doses of 8, 10, 12, 16, 20, 26, and 40 mg/kg at 24 h intervals between the injections (*n* = 11) (hereafter denoted as 8–40 mg/kg MPTP). In each control group, mice (*n* = 7–8 in each group) were subcutaneously injected with 0.9% NaCl according to the same regimens that were used to administer MPTP. The material for analysis was obtained 24 h after the last injection of MPTP or 0.9% NaCl.

At 23.5 h after the last injection of MPTP at 8–10 mg/kg and 8–40 mg/kg, or 0.9% NaCl, motor activity was reassessed in the open field test in mice. At 24 h after the last injection of MPTP, the mice were decapitated under isoflurane anesthesia (Baxter, Deerfield, IL, USA), and the brains were removed and cut along the middle sagittal plane. The striatum and SN were isolated using a dissecting microscope (Leica M60, Wetzlar, Germany), as described earlier [21]. The obtained samples of striatum and SN were weighed, frozen in liquid nitrogen, and stored at −70 °C until the concentration of DA was determined by HPLC-ED.

#### 4.2.2. Preparation of a Substantia Nigra Cell Suspension and Staining of Dopaminergic Neurons After Modeling Parkinson’s Disease

To assess the gene expression of axonal transport proteins, a cell suspension of the SN was prepared, and DAergic neurons were stained according to the previously described protocol, which was slightly modified [32]. For this purpose, 24 h after 8–10 mg/kg and 8–40 mg/kg MPTP injections, as described above (*n* = 10 for each group), the mice were anesthetized with 2.4% isoflurane (Baxter, Deerfield, IL, USA) and decapitated, and the brain was extracted from the skull. Serial frontal sections of the brain were then prepared on a Leica VT1200S vibratome (Leica Biosystems, Wetzlar, Germany) with a thickness of 200 μm at the SN level, according to the mouse brain atlas (Allen Mouse Brain Atlas, available from mouse.brain-map.org): from −2.54 mm to −3.88 mm in the rostro-caudal direction relative to Bregma. SN tissue isolated from the right hemisphere of each section was frozen in liquid nitrogen and stored at −70 °C until RNA isolation. SN tissue isolated from the left hemisphere of each section was incubated in a 2 mg/mL papain solution (Sigma-Aldrich, St. Louis, MO, USA) prepared using DMEM (Gibco, Thermo Fisher Scientific, Waltham, MA, USA) for 30 min at 37 °C with constant stirring. To stop the enzymatic action of papain, fetal bovine serum (Gibco, Thermo Fisher Scientific, Waltham, MA, USA) was cooled to 4 °C and added to the tubes to a final concentration of 10% (*v*/*v*). The sections were then washed in Hank’s Balanced Salt Solution (Gibco, Thermo Fisher Scientific, Waltham, MA, USA) and centrifuged at 70× *g* twice for 30 s each time.

To stain DAergic neurons, the precipitate was incubated for 15 min in Hanks’ Balanced Salt Solution containing 50 nM GBR-BODIPY FL (an analogue of the DA reuptake inhibitor GBR12909 containing the fluorophore BODIPY^®^ FL, synthesized by the IBCh RAS Oxylipin Laboratory), followed by dissociation of SN vibratome sections by pumping the contents of the tube 15 times through pipette tips with a 0.5 mm tip, avoiding the formation of air bubbles. The resulting solution was incubated with 10 μm DRAQ5 (Abcam, Cambridge, UK) and 50 nM GBR-BODIPY FL for 15 min at 37 °C. The contents of the tube were then re-dissociated by pumping 15 times through pipette tips with a 0.5 mm tip, avoiding the formation of air bubbles. The volume of the stained cell suspension was brought to 500 μL using Hanks’ Balanced Salt Solution, and the suspension was filtered through a humidified 40 μL cell strainer (Falcon, Corning, Corning, NY, USA). The cells were then centrifuged for 5 min at 500× *g* and 4 °C. The supernatant was collected, and the precipitate was resuspended in 500 μL Hanks’ Balanced Salt Solution and stored at 4 °C until fluorescence-activated cell sorting (FACS).

RiboLock RNase inhibitor (Thermo Fisher Scientific, Waltham, MA, USA) was added to all solutions used to the concentration of 100 U/mL. The samples obtained were stored at −70 °C until analyzed by RT-PCR.

#### 4.2.3. Brain Fixation and Preparation of Frozen Samples for Subsequent Immunohistochemistry After Modeling Parkinson’s Disease

Semi-quantitative analysis of axonal transport protein content in DAergic neurons was carried out 24 h after the last injection using the regimens of 8–10 or 8–40 mg/kg MPTP or 0.9% NaCl (*n* = 4 in each group), as described above. The animals were anesthetized with chloral hydrate (Sigma-Aldrich, St. Louis, MO, USA), followed by intracardiac perfusion with 0.02 M phosphate-buffered saline (PBS) (pH 7.2–7.4, 37 °C) for 15 min and then with 4% paraformaldehyde (Sigma-Aldrich, St. Louis, MO, USA) in 0.1 M phosphate buffer (pH 7.2–7.4, 4 °C) for 15 min. The animal was then decapitated, and the brain was isolated and fixed by immersion in 4% paraformaldehyde for 12 h at 4 °C. Next, the brain was washed with 0.02 M PBS at room temperature 3 times for 10 min each time and placed into 20% sucrose (Sigma-Aldrich, St. Louis, MO, USA) in PBS for 24 h for cryoprotection. The brains were sectioned along the midsagittal plane and then frozen in hexane at −40 °C and stored at −70 °C until immunohistochemical staining.

### 4.3. High-Performance Liquid Chromatography with Electrochemical Detection

DA levels were measured in SN and striatum samples using HPLC-ED. To prepare the samples, tissue was homogenized using an ultrasonic homogenizer (UP100H, Hielscher Ultrasonics GmbH, Teltow, Germany) in 0.1 N HClO_4_ (Sigma-Aldrich, St. Louis, MO, USA) in a solution containing the internal standard 3,4-dihydroxybenzylamine hydrobromide (Sigma-Aldrich, St. Louis, MO, USA) at a concentration of 250 pmol/mL. The solution was then centrifuged at 20,000× *g* for 20 min. DA separation was performed on a ReproSil-Pur ODS-3, 4 × 100 mm, 3 μm pore diameter reversed-phase column (Dr. Majsch, Ammerbuch, Germany) at +30 °C and a mobile phase flow rate of 1 mL/min using an LC-20ADsp liquid chromatograph (Shimadzu, Kyoto, Japan). The mobile phase consisted of 0.1 M citrate-phosphate buffer, 0.3 mM sodium octanesulfonate, 0.1 mM EDTA, and 8% acetonitrile (all reagents from Sigma-Aldrich, St. Louis, MO, USA), pH 2.5. Electrochemical detector Decade II (Antec Leyden, Leyden, The Netherlands) equipped with a glassy carbon working electrode (+0.85 V) and an Ag/AgCl reference electrode was used. DA and internal standard peaks were identified by their release times in a standard solution. The analyte content was calculated using the internal standard method as the ratio of the peak areas of DA to the peak areas of these substances in the biological sample using LabSolutions software v 5.87 (Shimadzu, Japan). Striatal samples were normalized by tissue mass.

### 4.4. Fluorescence-Activated Cell Sorting

SN cells stained with DRAQ5 and GBR-BODIPY FL (DAergic neurons) were sorted using a BD FACSAria Fusion cell sorter (BD, Franklin Lakes, NJ, USA) at a pressure of 20–21 psi using a 100 μm diameter nozzle. The staining thresholds for DRAQ5 and GBR-BODIPY FL were established by analyzing an unstained cell suspension. The dyes used have non-overlapping emission spectra: DRAQ5: maximum at 697 nm; GBR-BODIPY FL: maximum at 511 nm. DRAQ5 was excited with a 640 nm laser and detected using a 670/30 nm band pass filter (DRAQ5 channel). GBR-BODIPY FL was excited with a 488 nm laser and detected with a 530/30 nm band pass filter (GBR-BODIPY FL channel). Distribution density histograms of all detected events (detected by the particle sorter) were then plotted and analyzed using the FlowJo V10.8.1 software (BD, Franklin Lakes, NJ, USA).

DAergic neurons contained in all suspensions were sorted into a tube containing 250 μL TRI-reagent (Sigma-Aldrich, St. Louis, MO, USA). After cell sorting was completed, the contents of the tubes were mixed on a vortex. The tube contents were frozen in liquid nitrogen and stored at −70 °C until RNA isolation and analysis by RT-PCR.

### 4.5. Real-Time PCR

Total RNA was isolated from sorted DAergic neurons in 1 mL of TRI-reagent according to the manufacturer’s instructions (Sigma-Aldrich, St. Louis, MO, USA). For this purpose, 750 μL of TRI-reagent was added to thawed samples. For better RNA precipitation, 1 μg of glycogen (Thermo Fisher Scientific, Waltham, MA, USA) was added to each sample. The concentration of total RNA in the samples was determined using a NanoDrop 8000 spectrophotometer (Thermo Fisher Scientific, Waltham, MA, USA). Total RNA was treated with DNase I, RNase-free (Thermo Fisher Scientific, Waltham, MA, USA) to remove residual genomic DNA according to the manufacturer’s recommendations. Thereafter, 0.15 μg of RNA was subjected to reverse transcription to synthesize cDNA. Reverse transcription was performed using a Maxima H Minus First Strand cDNA synthesis kit with random hexamer primers according to the manufacturer’s instructions (Thermo Fisher Scientific, Waltham, MA, USA). The concentration of cDNA was measured using a NanoDrop 8000 spectrophotometer. Real-time PCR was performed on a QuantStudio 12k Flex thermal cycler (Applied Biosystems, MA, USA) using a qPCRmix-HS SYBR+LowROX reaction mixture (Evrogen, Moscow, Russia) and oligonucleotide primers (Evrogen, Moscow, Russia) (Table 1). The reaction was performed using 500 ng of cDNA.

Gene expression was evaluated using the 2^−ΔΔCt^ method. *Cyc1* was selected as an endogenous control because its expression remained more stable in a mouse model of PD, showing less fluctuation than *Rpl13* [61]; the latter had previously been recommended as an optimal housekeeping gene for gene expression studies in neurodegenerative diseases [62]. Formulas (1) and (2) were used to calculate ΔΔCt:

∆∆Ct = (∆Ct(sample) − ∆Ct(control mean))(1)

where

∆Ct = (Ct(gene) − Ct(*Cyc1*)) (2)

The results were calculated as the geometric mean of the group [63] and presented as the fold change compared with the control. 2^−ΔΔCt^ of the control group is taken as 1.

The gene names were taken from the National Library of Medicine’s GenBank database (https://www.ncbi.nlm.nih.gov/genbank, accessed on 25 May 2025).

### 4.6. Preparation of Frontal Sections of the Midbrain, Including the Substantia Nigra, for Immunohistochemistry

Twelve-μm-thick serial frontal sections of the brain, including the SN (from −2.54 to −4.04 relative to Bregma according to the Allen Mouse Brain Atlas), were prepared on a Leica CM1950 cryostat (Leica Microsystems GmbH, Wetzlar, Germany). The content of the following axonal transport proteins was analyzed in this study: β3-tubulin, α-tubulin, KLC1, Kif5B, dynactin 1 and tau protein (Table 2). Every 5th SN section was mounted on slides for subsequent immunohistochemistry for each protein of interest and tyrosine hydroxylase as a marker of DAergic neurons. Some sections were used for background fluorescence value determination. They were subjected to all immunohistochemistry procedures except for incubation with primary antibodies. Thus, 10–11 sections from the rostral to the dorsal parts of the SN, with 96 μm distance between the sections, were used for subsequent immunohistochemistry for each protein of interest. On each slide, there were brain sections from both control mice and PD model animals.

### 4.7. Immunohistochemistry of Frontal Sections with the Substantia Nigra

Frontal sections with the SN were sequentially incubated with: (i) 1% sodium dodecyl sulfate (Sigma-Aldrich, St. Louis, MO, USA) in PBS for 5 min; (ii) 3% bovine serum albumin (BSA) (Sigma-Aldrich, St. Louis, MO, USA) and 0.3% Triton X-100 (Sigma-Aldrich, St. Louis, MO, USA) in PBS for 1 h; (iii) sheep antibodies against tyrosine hydroxylase together with one of the antibodies against a protein involved in axonal transport, except β3-tubulin (Table 2), 1% BSA, and 0.1% Triton X-100 in PBS for 20 h; (iv) donkey anti-rabbit IgG, labeled with Alexa Fluor 555 (1:1000, A32794, Invitrogen, Thermo Fisher Scientific, Waltham, MA, USA) in PBS for 2 h; and (v) donkey anti-sheep IgG, labeled with Alexa Fluor 633 (1:1000, A21100, Invitrogen, Thermo Fisher Scientific, Waltham, MA, USA) in PBS for 2 h. After each incubation, except the second, the sections were washed in PBS for 30 min. Frontal sections with the SN were mounted in a mounting medium with DAPI (Abcam, Cambridge, UK).

For detection of β3-tubulin on frontal sections of the SN, the sections were sequentially incubated with: (i) 1% sodium dodecyl sulfate (Sigma-Aldrich, St. Louis, MO, USA) in PBS for 5 min; (ii) 36 μg/μL blocking reagent (Vector Labs, Newark, CA, USA), 3% BSA, and 0.3% Triton X-100 in PBS for 1 h; (iii) protein concentrate 80 μg/μL (Vector Labs, Newark, CA, USA), 3% BSA, and 0.3% Triton X-100 in PBS for 20 min; (iv) sheep anti-tyrosine hydroxylase antibody together with mouse anti-β3-tubulin (Table 2), 1% BSA, and 0.1% Triton X-100 in PBS for 20 h; (v) donkey anti-mouse IgG, labeled with Alexa Fluor 555 (1:1000, A32773, Invitrogen, Thermo Fisher Scientific, Waltham, MA, USA), supplemented with protein concentrate (80 μg/μL) in PBS for 2 h; and (vi) donkey anti-sheep IgG, labeled with Alexa Fluor 633 (1:1000, A21100, Invitrogen, Thermo Fisher Scientific, Waltham, MA, USA) in PBS for 2 h. After each incubation, except the second and third, the sections were washed in PBS for 30 min. Frontal sections with the SN were mounted in a mounting medium with DAPI.

### 4.8. Microscopy

SN frontal sections after immunohistochemistry for proteins involved in axonal transport were studied using a Plan-Apochromat 20×/0.8 M27 objective (Carl Zeiss AG, Jena, Germany) in a Zeiss LSM 880 confocal microscope (Carl Zeiss AG, Jena, Germany) (Core Center of the Institute of Developmental Biology RAS). Alexa Fluor 633 fluorescence, together with DAPI and Alexa Fluor 555, was detected in different channels. For each SN, a 4 × 3 mosaic was created with a 10% overlap between images.

For each control-experiment pair, individual photography conditions were selected according to laser intensity, which were then applied to all sections of the pair, as well as to the sections that were incubated without the primary antibodies.

### 4.9. Semi-Quantitative Assay of the Content of Axonal Transport Proteins in Dopaminergic Neurons

**Figure 8:**
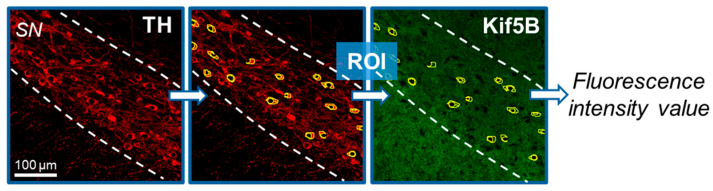
Schematic representation of the selection of the region of interest (ROI) for semiquantitative assessment of the levels of proteins involved in axonal transport (here, for example, Kif5B, green) in the cell bodies of tyrosine hydroxylase (TH)-immunoreactive neurons (red) in the substantia nigra (SN). Scale bar = 100 μm. Kif5B, kinesin family member 5B; ROI, region of interest; SN, substantia nigra; TH, tyrosine hydroxylase.

For the semi-quantitative determination of axonal transport protein content in the DAergic neurons (cell bodies), each tyrosine hydroxylase-immunoreactive neuron was outlined, excluding the nucleus region, as a region of interest (ROI) for use in FIJI (software available online: https://imagej.net/software/fiji/downloads, accessed 20 January 2025) (Figure 8). The resulting ROIs from the cell bodies of DAergic neurons were then overlaid with a channel containing the signal from axonal transport proteins in the original photographs, and the fluorescence intensity was determined using the FIJI program. SN areas in the sections incubated without the primary antibodies were used as background.

Fluorescence intensity was calculated as the difference between the mean signal intensity and the mean background intensity.

### 4.10. Statistical Analysis

Statistical analysis was performed using GraphPad Prism 9 software (GraphPad Software, San Diego, CA, USA). The normality of the distribution for each group was evaluated with the Shapiro–Wilk test. To compare two unrelated groups, either the *t*-test or the Mann–Whitney test was used, depending on the distribution type. For pairwise comparisons, either the paired *t*-test or the Wilcoxon test was used. The results are presented as mean ± SEM or as mean with interquartile range. Differences were considered significant at *p* ≤ 0.05.

## 5. Conclusions

The present findings indicate that, in a model of preclinical stage PD, dopaminergic neurons of the substantia nigra exhibit bidirectional changes in the expression of genes encoding motor proteins, microtubule components, and regulatory proteins of axonal transport. The increased expression of *Kif5b* and the elevated level of kinesin heavy chain (Kif5B), which mediates anterograde transport of organelles and vesicles, likely represent a compensatory response to early neurodegenerative alterations. As nigrostriatal degeneration progresses to the model of the clinical stage of PD, the expression of most analyzed genes declines and Kif5B levels decrease, likely reflecting the exhaustion of compensatory mechanisms that sustain anterograde axonal transport.

## Figures and Tables

**Table 1:** Oligonucleotide primers used for real-time PCR.

Gene	Protein	Forward Primer	Reverse Primer
*Cyc1*	Cytochrome C1	5′-GCGGGGGGCCAGGGGGAAGTTGT-3′	5′-GCCAGGAGTGAGCAGGGAAAATAC-3′
*Kif5b*	Kinesin family member 5B	5′-TCGTGTGTGTGTTTTCCAGTCAAGCA-3′	5′-CTCCATCGTGTGTGGTGGTGTCTTCC-3′
*Klc1*	Kinesin light chain 1	5′-GGGCAAGTACGAGGAGGAGGTGTG-3′	5′-GTGCGCGCGGGTGAGAATC-3′
*Mark1*	Microtubule affinity regulating kinase 1	5′-AGAGAGACAGCAGCAGCCTTACAGAGAGAT-3′	5′-GTGGGGCCAGAGAGGTTGTTGACATAGA-3′
*Mapt*	Microtubule-associated protein tau	5′-CACCCCCCATCCCTACCAACAACA-3′	5′-TCTGCAGGCAGGCGGCTCTTACTA-3′
*Tubb3*	β3-tubulin	5′-CTGTGTCCGCGCTGCCTGCCTTTTTTTTC-3′	5′-AGTTGCCGCCGCTGGGGGGGGTCTA-3′
*Tuba1a*	α-tubulin	5′-GGGGGGGAACTGGCTCTCTGG-3′	5′-GGGGGGGGCTGGGGGTAAATGG-3′
*Dynll1*	Dynein cytoplasmic light chain 1	5′-GGCCCCCATATCAAGAAGAAGGAGGAGTT-3′	5′-TGACCCCCAGGTAGAAGTAGTAGTAGATGAAG-3′
*Dync1i1*	Dynein cytoplasmic 1 intermediate chain 1	5′-GGACCACACGAAGCACACAACAA-3′	5′-GTCCACGCAGGCAGGCAAAGAGAG-3′
*Dctn1*	Dynactin 1	5′-GCCGCCAGAGACTTTTGAT-3′	5′-GCAGCAGCACCAGGACAC-3′

**Table 2:** Primary antibodies used for immunohistochemistry.

Protein	Host	Dilution	Incubation Condition	Manufacturer
α-tubulin	Rabbit	1:100	20 °C 20 h	PA5-19489, Invitrogen, Thermo Fisher Scientific, Waltham, MA, USA
β3-tubulin	Mouse	1:300	20 °C 20 h	ab7751, Abcam, Cambridge, UK
Dynein intermedial chain	Rabbit	1:100	4 °C 48 h	ab171964, Abcam, Cambridge, UK
Dynactin 1	Rabbit	1:300	4 °C 48 h	PA5-21289, Invitrogen, Thermo Fisher Scientific, Waltham, MA, USA
Kif5B	Rabbit	1:200	20 °C 20 h	ab167429, Abcam, Cambridge, UK
KLC1	Rabbit	1:300	20 °C 20 h	ab174273, Abcam, Cambridge, UK
Tau protein	Rabbit	1:300	20 °C 20 h	314 002, Synaptic Systems, Göttingen, Germany
Tyrosine hydroxylase	Sheep	1:700	−	ab1542, Sigma-Aldrich, St. Louis, MO, USA

## Data Availability

The original contributions presented in this study are included in the article. Further inquiries can be directed to the corresponding author.

## Abbreviations

The following abbreviations are used in this manuscript:

Akt	Serine/threonine protein kinase B
BSA	Bovine serum albumin
DA	Dopamine
DAergic	Dopaminergic
GSK-3β	Glycogen synthase kinase-3β
FACS	Fluorescence-activated cell sorting
Kif5B	Kinesin family member 5B
KLC1	Kinesin light chain 1
MPTP	1-methyl-4-phenyl-1,2,3,6-tetrahydropyridine
PBS	Phosphate-buffered saline
PD	Parkinson’s disease
ROI	Region of interest
SN	Substantia nigra
